# Digital Health Technology Compliance With Clinical Safety Standards In the National Health Service in England: National Cross-Sectional Study

**DOI:** 10.2196/80076

**Published:** 2025-10-31

**Authors:** Youssof Oskrochi, Elliott Roy-Highley, Keith Grimes, Sam Shah

**Affiliations:** 1 Global Business School for Health University College London London United Kingdom; 2 Bayes Business School University of London London United Kingdom

**Keywords:** digital health technologies, clinical safety standards, DCB0129, DCB0160, NHS England, health IT systems, patient safety, organizational compliance, clinical risk, clinical safety, clinical risk management, regulation, artificial intelligence, AI

## Abstract

**Background:**

To be authorized for use in the National Health Service (NHS) in England, digital health technologies (DHTs) must meet 2 mandatory clinical risk management standards, Data Coordination Board (DCB) 0129 and 0160, demonstrating that risks from design and use have been assessed and mitigated. NHS organizations must not procure a DHT without DCB0129 assurance and must not deploy one without DCB0160 assurance. Despite legal requirement, no public data exist on how many DHTs are in use in the NHS or how many are assured.

**Objective:**

This study aimed to determine the number of DHTs in use in the NHS in England and assess their assurance status against mandated clinical safety standards.

**Methods:**

In early 2025, 239 NHS organizations in England received a freedom of information notice requesting information on the number of DHTs they were using and their assurance against DCB0129 and DCB0160 standards.

**Results:**

Of the 239 NHS organizations, 204 (85.4%) responded, of which 178 (87.3%) provided full or partial data, covering 14,747 DHT deployments. The mean number of deployed DHTs per organization was 82.8 (SD 146.1; 95% CI 61.4-104.3) with substantial variation between NHS provider trusts (mean 107.1, SD 161.1; 95% CI 79.8-134.3), ambulance trusts (mean 13.0, SD 8.2; 95% CI 7.6-18.4), and integrated care boards (mean 8.1, SD 16.0; 95% CI 2.8-13.5). Overall organizational compliance rates were low, with a median of 25.6% (IQR 7.8%-55.7%) deployed DHTs being fully assured; for NHS provider trusts compliance was lower at 24.5% (IQR 8.1%-50%). A total of 13 (6.4%) of the 204 organizations reported that all their DHTs were fully assured, while 16 (7.8%) reported that none were assured. Across all DHTs with reported assurance data, 17.3% (95% CI 16.6%-18.1%) were fully assured against both standards, 13.3% were partially assured against one standard, and 70.1% (95% CI 69.1%-71.1%) had no documented assurance.

**Conclusions:**

This is the first study to quantify both the scale of DHT deployment in NHS organizations in England and the extent of compliance with mandatory safety standards. More than 10,000 DHTs currently in use lack documented assurance against clinical safety standards. In a typical NHS trust, 3 out of 4 digital tools influencing patient care do not demonstrate compliance with minimum legal or clinical safety requirements. These findings raise significant concerns about the risks posed to patients by these technologies; the capacity of organizations to assess and mitigate them; and the legal ramifications of when, not if, harm occurs. Crucially, failure to assure digital technologies poses a significant risk to one of the core ambitions of the NHS 10-Year Health Plan for England; safely transitioning from analogue to digital care models. These findings are unlikely to be unique to the NHS and should prompt health care systems worldwide to assess the risks posed by their DHT deployments.

## Introduction

### Background

The use of technology in health care features prominently in the National Health Service (NHS) Long Term Plan [1] and is one of the “3 big shifts” of the 10-Year Health Plan for England: Fit for the Future [2]. Digital transformation promises to serve as a vehicle through which health care organizations, many of which are struggling to meet demand, may improve outcomes, enhance the quality of care, reduce costs, and increase productivity.

As a result, digital transformation in the NHS has been heavily promoted, with virtual consultants, apps, patient portals, and wearables now being far more common than even 5 years ago [3].

However, no one actually knows how many digital technologies are being used in the delivery of care. As the use of digital technologies increases, patients and organizations are increasingly exposed to new risks, the impact of which spans organizational pressures, care quality, and patient safety. These risks are not hypothetical either; identification of the QRISK2 calculator code-mapping error required clinical rereview of 7 years’ worth of patients who were incorrectly scored [4], while more serious instances of IT system failures at NHS hospitals have resulted in patient deaths [5].

The safety of digital health technology (DHT) in the NHS is primarily governed by 2 standards published by the Data Coordination Board (DCB): DCB0129 [6] and DCB0160 [7]. These standards fall under Section 250 of the Health and Social Care Act 2012 and therefore are legal requirements for all digital systems used in health and adult social care settings in England.

The standards apply to “health IT systems” and are defined as any “product [software, hardware, or combination] which provides electronic information for health and social care purposes” [8]. This includes software and devices used for clinical and nonclinical (eg, procurement and clerical) purposes and applies to both medical and nonmedical devices as defined by the Medical Device Regulations 2002.

More recently, NHS Digital published step-by-step guidance [9] on the applicability of the standards to “digital products” and included a clarifying statement that eligible technologies are those “used to influence, support, manage the real-time or near-real-time direct care of patients/service users.” It should be noted that the requirement to impact “real-time or near-real-time direct care” is not present in the original specification [6].

In this paper, we use the term DHT to encompass all products falling within the scope of the digital clinical safety standards in line with terminology used by the World Health Organization [10], Medicines and Healthcare products Regulatory Agency [11], US Food and Drug Administration [12], and National Institute for Health and Care Research [13].

DCB0129 sets out the clinical risk management standards that manufacturers of DHTs must adhere to during the design and development of their products, evidencing the governance and processes they have undertaken to identify, assess, and mitigate risks. This standard ensures manufacturers have not only mitigated clinical risks identified in the development of the product but also continue to do so following product changes or in light of clinical incidents reported to the manufacturer.

DCB0160 sets out similar standards but for organizations that seek to deploy DHTs, requiring them to identify the risks the system may pose when implemented, during its use, when updated, and when decommissioned. This standard requires organizations to carry out a structured risk assessment, considering the severity of potential harm and the mitigations required to limit that harm for the use of the digital product *in the circumstances of their own specific deployment.*

These assessments must be carried out by qualified clinical safety officers (CSOs) who are clinical staff trained in digital safety.

Therefore, while a product may hold only one DCB0129 clinical safety case, which is reusable across deployments, a unique DCB0160 assessment needs to be carried out by every organization implementing that product. Compliance with the standards invariably results in the production of a clinical safety case, which is the sum of evidence demonstrating the implementation and outputs of the clinical risk management process. The clinical safety case report (CSCR) is the summary of that process, providing a coherent argument and presenting evidence that the product has been rendered safe by the manufacturer (DCB0129) and the deploying organization (DCB0160). Together, DCB0129 and DCB0160 are intended to create a critical safety chain across the life cycle of the product, ensuring that at each milestone, there is a clear assessment of the risks and robust mitigations in place to limit them [14].

There is no central mechanism by which compliance is monitored or reported. Although DCB0129 forms part of NHS England’s Digital Technology Assessment Criteria framework [15] for digital products, each deploying organization is ultimately responsible for ensuring it is compliant with legislation and the information standards.

The lack of monitoring and reporting means that there is currently no information on the number of DHTs used in the NHS or compliance rates with the legislated standards.

### Aims and Objectives

The aim of this study was to explore and quantify compliance with clinical risk management standards DCB0129 and DCB0160 in NHS organizations in England.

To achieve this, we sought to quantify the following:

1. The total number of DHTs used by NHS organizations
2. The number of DHTs fully assured (having both DCB0129 and DCB0160 CSCRs)
3. The number of DHTs partially assured (holding either DCB0129 or DCB0160 CSCR)
4. The number of DHTs that are not assured (holding no CSCR)

## Methods

### Study Design

Between February and March 2025, we undertook a cross-sectional study of NHS organizations in England using freedom of information (FoI) requests to obtain data on organizational compliance with clinical safety standards.

### Setting and Participants

The NHS Provider Directory [16] and the NHS System Directory [17] were used to generate a list of all NHS trusts and integrated care boards (ICBs) in England. Each entry on the list was manually checked and updated to ensure it reflected the latest information. For each entry, the correct contact details for FoI requests were also retrieved and stored.

### Data Sources

The Freedom of Information Act 2000 [18] makes provision for the disclosure of information held by public authorities (such as NHS organizations) that is not already publicly available through submission of a valid FoI request. Public authorities have up to 20 working days to respond to requests, seek extensions, or refuse requests [19].

A correctly formatted FoI request (Multimedia Appendix 1) was then sent to all identified participants.

Any responses that claimed exemptions under the legal framework of the act were challenged via the internal review process of the organization, as permitted under the same act. If the exempting organization later reversed its decision, the date on which its initial refusal was sent was still used as the date on which it responded to the FoI.

### Outcomes

We used the data collected via FoI request to calculate the following outcome measures:

1. Total number of DHTs deployed in NHS organizations in England
2. The proportion of DHTs deployed by an organization that were fully assured (organizational compliance rate).
3. Proportion of DHTs in use in the NHS that were fully, partially, or not at all assured against the clinical safety standards (DHT assurance status).

### Data Analysis

Although the legal time limit for FoI requests is 20 working days from the first notice, the number of regulatory actions (due to FoI delays) being undertaken by the Information Commissioner’s Office against NHS trusts suggested that many requests may be delayed [20]. Therefore, responses were accepted until June 2025.

Where organizations responded ambiguously with approximate numbers (eg, approximately 300), that value was taken as stated. When the response was with a “greater than or less than” estimate (eg, “more than 100” or “<10”), a conservative approach to interpretation was taken with the next valid number used (eg, “more than 100” was recorded as 101 and “<10” was recorded as 9).

If an organization was unable to provide a value for the number of digital health systems (question 1) but provided responses for all other questions, then a value equal to the sum of questions 2 to 4 was used as a proxy for question 1. This would almost certainly be an underestimate, as it fails to include those systems that would qualify but do not have accessible safety cases.

Where organizations provided partial responses, for example, provided an answer to questions 1 (total DHTs) and 2 (count of fully compliant systems) but not for any other question, then this was recorded as providing “partial data.”

All responses (quantitative and qualitative) were recorded in Microsoft Excel. Quantitative data were analyzed using RStudio (version 2024.12.1; Posit PBC).

Results were further broken down by organization type. Where appropriate and sufficient data were available, summary statistics, such as means (including SDs and CIs), medians (with IQRs), and ranges, were calculated.

### Bias

Bias was not deemed likely to have a significant impact on our study due to the design and methods used. We sampled all NHS organizations in the NHS directory, thus minimizing sampling bias. The use of the FoI mechanism, a statutory instrument, meant that organizations were compelled to reply, thus minimizing response bias, and the objective and quantifiable nature of the outcomes requested meant that the risk of information bias was limited.

### Ethical Considerations

Our unit of analysis was organizations; therefore, many ethical and privacy considerations regarding human participants were not applicable to our study. Nevertheless, we sought and received ethics approval from the University College London Research Ethics Committee (30–IHIREC), and steps were taken to ensure that only organizations, rather than individuals, were represented in our data.

### Reporting

The study was reported in accordance with STROBE (Strengthening the Reporting of Observational Studies in Epidemiology) guidelines for cross-sectional studies [21].

## Results

### Response Rate

In early 2025, a total of 239 NHS organizations were contacted (Multimedia Appendix 2). These included NHS trusts (foundation and nonfoundation, including ambulance trusts) and ICBs. By June 2025, a total of 204 (85.4%) organizations had responded.

In total, 36 organizations initially claimed exemptions under the act. Exemptions were claimed partially (for some questions) or fully (for all questions). After the challenge and internal review, 13 (36.1%) organizations provided answers: 6 (46.2%) fully and 7 (53.8%) partially.

### Completeness of Response

There was variation in the completeness of responses. Out of 204 organizations, 143 (70.1%) responded fully, 36 (17.6%) responded partially, and 25 (12.3%) responded but did not provide values (Table 1); instead, they either stated they “didn’t hold” the information or claimed exemptions under the act.

A total of 4.9% (10/204) of the organizations provided ambiguous responses for question 1 (total number of DHTs deployed) and were recorded as per the protocol outlined in the Methods section.

There were no ambiguous responses to the compliance questions (questions 2 and 3); as a result, the impact of the ambiguous responses to question 1 on compliance assessments was likely to be negligible.

**Table 1:** Response data and assessments of completeness of responses from a cross-sectional survey on the number of digital health technologies deployed by all organizations in the National Health Service (NHS) in England between March 2025 and June 2025.

Organization type	FoI^a^ response completeness				Did not respond, n (%)
	Full data provided, n (%)	Partial data provided, n (%)	No data provided, n (%)		
NHS provider trusts^b^ (n=187)	104 (55.6)	31 (16.6)	19 (10.2)	33 (17.6)	
Integrated care boards (n=42)	31 (73.8)	4 (9.5)	6 (14.3)	1 (2.4)	
Ambulance trusts (n=10)	8 (80)	1 (10)	0 (0)	1 (10)	
All (n=239)	143 (59.8)	36 (15.1)	25 (10.5)	35 (14.6)	

^a^FoI: freedom of information.

^b^Foundation and nonfoundation trusts with acute and/or mental health and community functions.

### DHT Deployments

In total, 178 (87.3%) of the 204 organizations responded to question 1, reporting a combined total of 14,747 active DHT deployments between them.

It is critical to emphasize that the DHT count represents deployments rather than unique products, meaning the same product will be represented multiple times in our data, commensurate with the number of times it has been deployed. This approach reflects the requirement that each organization must conduct its own DCB0160 safety case regardless of whether other organizations have already assessed the same product.

Ambiguous responses (n=10) accounted for 1746 DHTs, representing 11.84% (1746/14,747) of the total number of DHTs recorded. Of these, 4 organizations covering 942 (6.39%) DHTs responded to question 1 only (total DHT number) and did not provide adequate information for compliance assessments.

**Figure 1:**
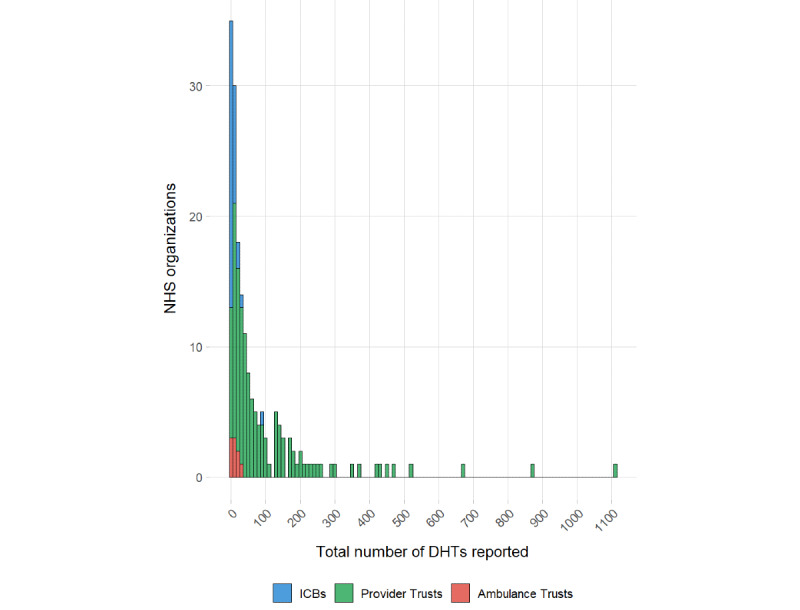
Histogram of total reported digital health technology (DHT) numbers by organization type from a cross-sectional survey of all National Health Service (NHS) organizations in England between March 2025 and June 2025. ICB: integrated care board.

The number of DHTs deployed by organizations was highly varied (Figure 1), with a large difference between the calculated mean (82.8) and median (27.5). A breakdown by organization type is presented in Table 2.

Among 134 NHS provider trusts, 21 (15.7%) organizations reported that they used 10 or fewer DHTs, with 3 (2.2%) reporting that they only used 3 DHTs across their entire organization.

ICBs reported the lowest number of DHTs with a median number of 3 (IQR 1-7.5) and a mean of 8.1 (SD 16; 95% CI 2.8-13.5), while NHS provider trusts reported the highest number of DHTs with a median of 49 (IQR 18.2-134.2) and a mean of 107.1 (SD 161.1; 95% CI 79.8-134.3). A total of 4 ICBs reported that they did not use any DHTs.

**Table 2:** Summary statistics of the number of deployed digital health technologies (DHTs) by organizations in the National Health Service (NHS) in England from a cross-sectional survey of all NHS organizations in England between March 2025 and June 2025.

Organization type	Organizations (n=178), n (%)	Total DHT deployments, (n=14,747), n (%)	Point estimate (SD; 95% CI)	Median (IQR)	Range
NHS provider trusts	134 (75.3)	14,345 (97.3)	107.1 (161.1; 79.8-134.3)	49.0 (18.2-134.2)	3-1115
Integrated care boards	35 (19.7)	285 (1.9)	8.1 (16.0; 2.8-13.5)	3.0 (1-7.5)	0-91
Ambulance trusts	9 (5.1)	117 (0.8)	13.0 (8.2; 7.6-18.4)	13.0 (4-18)	4-26
All	178 (100)	14,747 (100)	82.8 (146.1; 61.4-104.3)	27.5 (9-90.5)	0-1115

### Organizational Compliance With DCB Standards

Organizational compliance was evaluated by calculating the proportion of DHTs deployed that had both DCB0129 assurance and DCB0160 assurance, as determined by the presence of CSCR for each; 147 organizations provided data covering 9292 DHTs from which this could be calculated.

NHS provider trusts constituted most of these data, accounting for 9002 DHT deployments with an average of 81.1 per trust (SD 107.8; 95% CI 61.1-101.1). A full breakdown by organization type is provided in Table 3.

**Figure 2:**
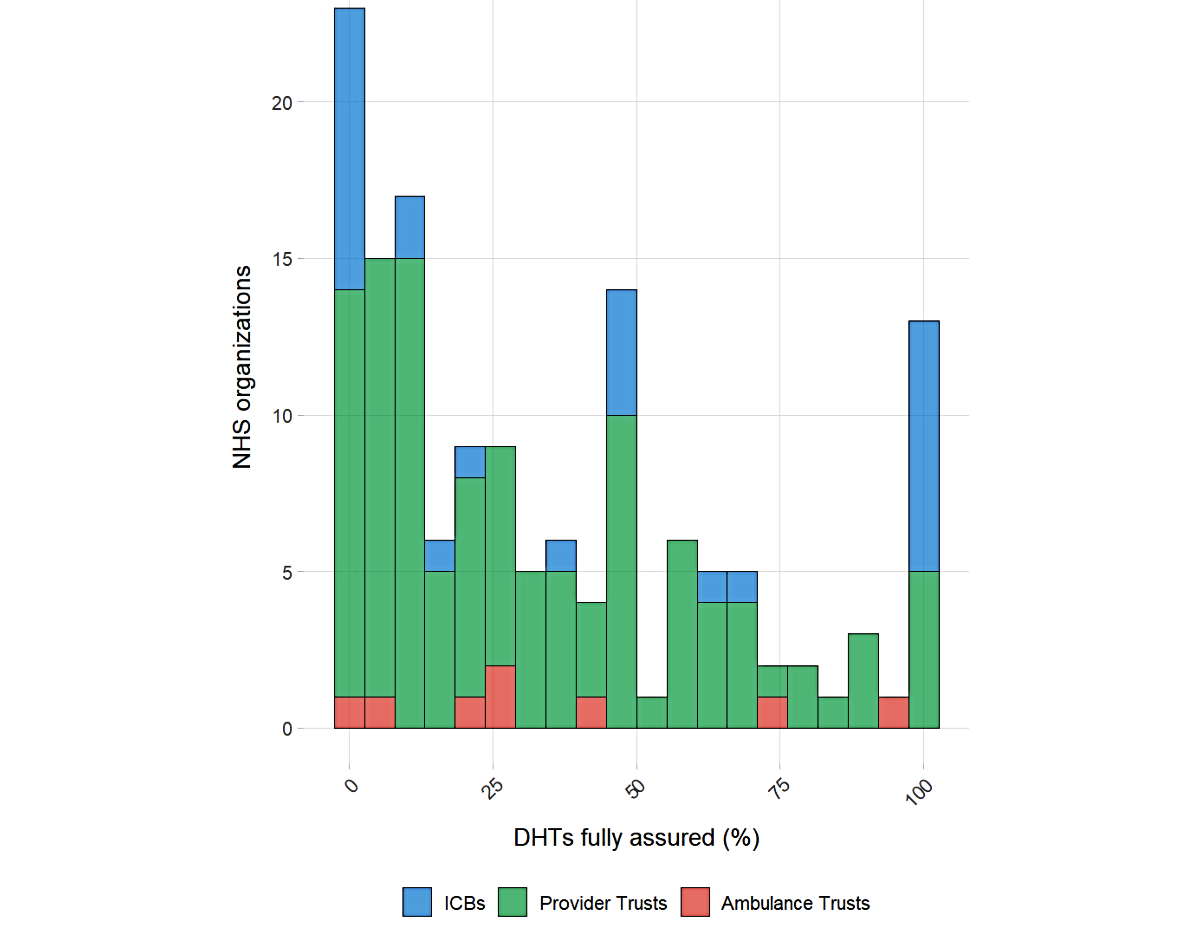
Histogram showing distribution of compliance rates with DCB0129 or DCB0160 clinical safety standards among National Health Service (NHS) organizations in England. Data are from a cross-sectional survey of all NHS organizations in England between March 2025 and June 2025. DHT: digital health technology; ICB: integrated care board.

Compliance rates had a moderately right-skewed distribution (skewness=0.74), indicating a clustering of compliance rates at the lower end of the scale (Figure 2).

Median compliance rates across all organizations were 25.6%, with the mean compliance rate being 34.7% (SD 31.8%; 95% CI 29.6%-39.9%; Table 4). For a typical (median) NHS trust, 24.5% (IQR 8.1%-50%) of the DHTs deployed were reported to have both DCB0129 and DCB0160 assurances.

**Table 3:** Summary statistics of the number of deployed digital health technologies (DHTs) by National Health Service (NHS) organizations that provided compliance data (n=147) from a cross-sectional survey of all NHS organizations in England between March 2025 and June 2025.

Organization type	Organizations (n=147), n (%)	Total DHT deployments, (n=9292), n (%)	Point estimate (SD: 95% CI)	Median (IQR)	Range
NHS provider trusts	111 (75.5)	9002 (96.9)	81.1 (107.8; 61.1-101.1)	42.0 (17-97.5)	3-671
Integrated care boards	28 (19)	186 (2)	6.6 (7.5; 3.9-9.4)	3.5 (1.8-8.2)	1-31
Ambulance trusts	8 (5.4)	104 (1.1)	13.0 (8.8; 6.9-19.1)	12.5 (4-19.2)	4-26
All	147 (100)	9292 (100)	63.2 (98.8; 47.2-79.2)	26.0 (9-71)	1-671

**Table 4:** National Health Service (NHS) organization compliance rates with the DCB0129 and DCB0160 clinical safety standards, as determined by the assurance status of deployed digital health technologies (DHTs). Data are from a cross-sectional survey of all NHS organizations in England between March 2025 and June 2025.

Organization	Organizations, n (%)	Mean (SD; 95% CI)	Median (IQR)	Range
NHS provider trusts	111 (75.5)	32.3 (28.7; 27-37.7)	24.5 (8.1-50)	0-100
Integrated care boards	28 (19)	43.8 (41.8; 28.3-59.2)	43.2 (0-100)	0-100
Ambulance trusts	8 (5.4)	35.7 (32.7; 13-58.3)	26.4 (15.5-50)	0-92.3
All	147 (100)	34.7 (31.8; 29.6-39.9)	25.6 (7.8-55.7)	0-100

In total, 13 (6.4%) of the 204 NHS organizations (n=8, 3.9% ICBs and n=5, 2.5% NHS provider trusts) reported full compliance for all the DHTs they had deployed. Moreover, out of 204, 12 (5.9%) organizations held a DCB0129 safety case for all DHTs deployed, and 3 (1.5%) held a DCB160 safety case for all DHTs deployed.

A total of 16 (7.8%) of the 204 organizations (n=9, 4.4% ICBs; n=6, 2.9% NHS provider trusts; n=1, 0.5% ambulance trust) reported that none of their DHTs had a digital clinical risk assessment recorded.

### Sensitivity Analysis for Organizational Compliance With DCB Standards

To assess the robustness of our compliance findings, we conducted 2 sensitivity analyses to determine whether outliers in DHT numbers or compliance rates impacted the results.

To determine the influence of extreme DHT values, we trimmed the dataset to exclude the top and bottom 5% (14/178) of the observations based on total DHT numbers deployed.

To determine the influence of extreme compliance rates, we excluded organizations with 0% compliance (ie, no DHTs fully assured; n=16) and 100% compliance (all DHTs assured; n=13).

In both analyses, the central tendency measures remained relatively stable compared to our primary analysis (Table 5) across all settings, suggesting that our findings are not driven by outliers in DHT number or compliance rates.

**Table 5:** Sensitivity analysis of organizational compliance rates for digital health technologies (DHTs) deployed by organizations in the National Health Service (NHS) in England, restricting data by total DHT number (middle 90%) and compliance rates (excluding 0% and 100% compliance). Data are from a cross-sectional survey of all NHS organizations in England between March and June 2025.

Organization type	Primary analysis					Sensitivity analysis								
	Organizations (n=147), n (%)	Mean (SD)	95%CI	Median (IQR)	Restricted by DHT number						Restricted by the compliance rate			
					Organizations (n=133), n (%)		Mean (SD)	95% CI	Median (IQR)	Organizations (n=118), n (%)		Mean (SD)	95% CI	Median (IQR)
NHS provider trusts	111 (75.5)	32.3 (28.7)	27-37.7	24.5 (8.1-50)	104 (78.2)		34.2 (28.7)	28.7-39.7	26.4 (9.4-53.6)	100 (84.7)		30.9 (24.9)	26-35.8	25.1 (9.3 - 50)
Integrated care boards	28 (19)	43.8 (41.8)	28.3-59.2	43.2 (0-100)	21 (15.8)		48.8 (39.1)	32.1-65.6	50.0 (13-100)	11 (9.3)		38.7 (20.7)	26.5-50.9	50.0 (18.3-50)
Ambulance trusts	8 (5.4)	35.7 (32.7)	13-58.3	26.4 (15.5-50)	8 (6)		35.7 (32.7)	13-58.3	26.4 (15.5-50)	7 (5.9)		40.8 (31.7)	17.3-64.3	27.8 (22.1-58.3)
All	147 (100)	34.7 (31.8)	29.6-39.9	25.6 (7.8-55.7)	133 (100)		36.6 (31.0)	31.3-41.9	27.8 (9.7-56)	118 (100)		32.2 (24.9)	27.7-36.7	26.2 (9.8-50)

### Assurance Status of All DHTs Currently Deployed by the NHS in England

Our sample covered a total of 14,747 DHT deployments currently in use across 204 NHS organizations in England.

The clinical risk assurance status of these was reported as follows:

- 17.3% (1612/9292; SD 0.39%; 95% CI 16.6%-18.1%) of the DHTs were fully assured with both DCB0129 and DCB0160 risk assessments having been undertaken. This was based on data from 151 organizations that provided responses to FoI questions 1 and 2, which covered 9292 DHTs.
- 8.7% (746/8560; SD 0.30%; 95% CI 8.1-9.3%) of the DHTs were partially assured, with only a DCB0129 risk assessment having been undertaken. This was based on data from 143 organizations that provided responses to FoI questions 1 and 3, which covered 8560 DHTs.
- 4.6% (415/9014; SD 0.22%; 95% CI 4.2-5.0%) of the DHTs were partially assured with only a DCB0160 risk assessment having been undertaken. This was based on data from 147 organizations that provided responses to FoI questions 1 and 4, which covered 9014 DHTs.
- 70.1% (5982/8536; SD 0.5%; 95% CI 69.1-71.1%) of the DHTs were not assured at all, with no risk assessment having been undertaken. This was based on data from 143 organizations that provided responses to all FoI questions and covered 8536 DHTs.

A breakdown of overall DHT assurance by organization type based on the aforementioned data is shown in Table 6.

**Table 6:** Summary statistics of the clinical safety assurance status of all digital health technologies (DHTs) deployed in the National Health Service (NHS) in England as a whole against both the DCB0129 and DCB0160 clinical safety standards. Data are from a cross-sectional survey of all NHS organizations in England between March 2025 and June 2025.

Organization type	Total DHTs (n=14,747), n (%)	Assurance (%)			
		Fully assured (SD; 95% CI)	Partially assured (SD; 95% CI)		Not at all assured (SD; 95% CI)
			DCB0129 only	DCB0160 only	
NHS provider trusts	14,345 (97.3)	16.7 (0.39; 16-17.5)	8.2 (0.30; 7.6-8.8)	4.7 (0.23; 4.2-5.1)	71.3 (0.50; 70.3-72.3)
Ambulance trusts	117 (0.8)	30.8 (4.53; 21.9-39.6)	11.5 (3.13; 5.4-17.7)	3.8 (1.89; 0.2-7.5)	53.8 (4.89; 44.3-63.4)
Integrated care boards	285 (1.9)	39.8 (3.59; 32.8-46.8)	35.5 (3.84; 28-43)	2 (0.90; 0.3-3.8)	15.6 (2.92; 9.9-21.3)
All organizations	14,747 (100)	17.3 (0.39; 16.6-18.1)	8.7 (0.30; 8.1-9.3)	4.6 (0.22; 4.2-5.0)	70.1 (0.50; 69.1-71.1)

## Discussion

### Principal Findings

Across 178 NHS organizations in England, 14,747 DHTs were reported to be deployed and currently in use by NHS provider trusts, ambulance trusts, and ICBs. NHS provider trusts naturally reported the largest number of DHT deployed, with an average of 107 per organization, although there was high variability.

Reported organizational compliance with clinical safety standards across NHS organizations was low, with, on average, only 34.7% of the deployed DHTs by each organization being fully assured against the DCB standards, falling to 32.3% when limited to NHS provider trusts only.

However, given the skewness of the data, the use of the median compliance figure may be more appropriate and suggests that, for a typical NHS trust, only 24.5% of its deployed DHTs are assured. This implies that more than 75% of the DHTs used in a typical NHS provider trust therefore do not meet the minimum clinical safety and legal requirements for use.

More concerning is the compliance status of DHTs deployed across the NHS. Only 17.35% (1612/9292) of the DHTs were fully assured, and a further 13.3% were partially assured (746/8560, 8.71% against DCB0129 only; 415/9014, 4.6% against DCB0160 only). More than 70% of deployed technologies had no documented evidence of safety assurance at all. Extrapolating this to all DHTs in our survey suggests that 10,470 deployments currently in use may be completely unassured.

The findings present a stark picture of digital clinical safety within NHS organizations in England. The public rightly places immense trust in the NHS to provide care that is not only effective but fundamentally safe [22]. This expectation extends to all tools used to deliver care, from medications to DHTs, yet our analysis profoundly challenges this trust.

### Unseen Risks and Unquantified Harm

More than 10,000 unassessed DHTs identified in our sample are necessarily a result of substandard procurement practice (not asking for DCB0129), organizational failings (lack of DCB0160), or “legacy debt.”

Legacy debt refers to the burden of historical software used in NHS trusts that has not been assured after many years against DCB0160 and was the subject of an advisory statement published by the Digital Health Networks CSO Council in October 2024 [23]. The CSO Council argued that for legacy systems, which have been operating without issue for many years, “the scale of clinical risk is likely lower.” We argue that while the risk to patient safety posed by unassured systems—regardless of their cause—is unknown, it should be a cause for significant concern. We base this assertion on historical and contemporary examples of digital failures discovered, in some instances, many years after deployment, which have caused significant harm, including deaths (Table 7).

**Table 7:** Historical and contemporary examples of harm due to IT system failures.

System	Fault	Time until fault discovered or acted on (y) and range	Harm or impact	Study
National breast screening IT system	Incompatibility between systems and failure of the “failsafe system.”	9 (2009-2018)	122,000 women affected	[24]
QRISK2 TPP	Code-mapping errors result in incorrect risk stratification.	7 (2009-2016)	260,000 to 270,000 individuals affected	[4,25]
Cerner EHR^a^	Clinical orders sent to incorrect locations, therefore not fulfilled (eg, referrals and medications).	2 (2020-2022); ongoing from March 2024	149 patient harm events; possibly more	[26]
Horizon IT system	Primarily, software bugs are compounded by corporate culture, leading to incorrect accounting.	20 (1999-2019)	More than 700 subpostmasters convicted of fraud	[27,28]

^a^EHR: electronic health record.

Digital systems are particularly prone to hidden and latent failures.

Hidden failures occur when individuals, processes, or organizations do not have the capability (knowledge, awareness, or otherwise) to identify or investigate faults. Harm that occurs may therefore be misattributed to other parts of the care pathway that are more easily interrogated. This will likely become an even greater concern with the rise of “black box” products, such as those incorporating artificial intelligence (AI).

Latent failures are flaws that remain dormant until specific conditions trigger them, potentially leading to catastrophic harm that can scale exponentially [29].

This situation is compounded by the well-documented underreporting of software issues in health care [30,31], where staff may hesitate to report problems due to fear of consequences [32], lack of time, or a culture that rewards “working around” issues [33,34]. Thus, the absence of evidence of harm should not be mistaken for evidence of safety; due diligence must be exercised.

The absence of a DCB0160 safety case for 78% (11,502/14,747) of the DHTs, whether new or legacy, is particularly concerning.

Servicing this “compliance debt” requires significant resource prioritization and commitment from the NHS.

Although we recognize that many NHS organizations are perpetually struggling with a multitude of competing service delivery demands in a fiscally constrained environment, we do not believe that these challenges should take precedence over effective clinical safety work. Prioritizing operational and fiscal pressures or failing to implement and execute proper governance and assurance processes often comes at the expense of safety, something which has repeatedly been shown to result in tragic and often avoidable harm [35-37].

Ultimately, and as stated in the standards, “top management” has overall accountability for the DHTs that are deployed by their organization, and it is likely that if an injury claim arises due to the harm caused by a DHT, claimants could use the lack of compliance to demonstrate reckless behavior on the part of the NHS organization that deployed it.

### Interpreting Low Compliance

Although we did not set out to explicitly identify the causes of low compliance, additional qualitative responses submitted by responding organizations provided valuable insights. Differing interpretations of applicability, deficits in procurement processes, a reactive approach to clinical safety, and locally variable implementation of the standards were recurring themes offered as potential reasons for low compliance rates (Textbox 1).

In our opinion, these responses are substandard—definitions are clearly written and stated, assurance has been legally required since 2012, and the purpose of the clinical standards is that they are a proactive process and not a reactive one in response to “serious” incidents. We further suggest that it is highly unlikely that systems deployed since 2012 have not received any updates, qualifying them for assurance reviews.

**Selection of free-text responses to freedom of information questions.**

- “Health IT is often more narrowly defined as systems that directly manage clinical care, such as electronic health records or prescribing tools.”
- “This [DCB0129] is not requested as part of the IT procurement process but suppliers should provide this by default. However, it is not recorded.”
- “Legacy systems are reviewed if serious incident occurs, or they undergo development, at which point clinical safety work is undertaken.”
- “Clinical safety process did not commence until 2022 and it was decided that only new systems or upgrades would come under the process.”
- “The Trust have implemented some Health IT systems which are clinical, where the supplier has not provided DCB0129 documentation. This is either because, the clinical system predates DCB0129, or was poorly understood by suppliers and no dcb0129 documents were available at the time of purchase, despite requesting them. In this situation, systems are assessed when they are due for an upgrade.”

Of the DHTs in our study with available compliance data, 74.7% lacked evidence of DCB0129 assurance. Extrapolating this finding suggests that approximately 11,000 of all reported deployments may be similarly noncompliant. This highlights a substantial gap in basic safety assurance during procurement, where DCB0129 is either not requested as part of the procurement processes (as evidenced by free-text responses) or, when requested, not logged or recorded effectively, as suggested by respondents who claimed they do not hold such information. This suggests that the foundational step in the due diligence process—requesting, receiving, reviewing, and documenting safety assurance—may not be occurring consistently. Failure to complete or record this initial step should justify delaying procurement, let alone deployment; yet our findings indicate that approximately 75% of the procured and deployed products lack documented evidence of having completed the first stage of the safety chain.

There also appears to be significant confusion and variability between NHS organizations in which digital technologies fall under the scope of the standards. ICBs reported the lowest number of DHTs, consistent with their function. However, NHS provider trusts provided wide estimates of the number of DHTs deployed; some reported as few as 3 DHTs, while others reported more than 1000 DHTs deployed. Free-text responses reveal that some organizations are applying narrower definitions than the standards, with 1 claiming that only their “five core EPR systems” fell under the remit of the Health IT system, while others claimed narrower definitions than those in the standards (Textbox 1). This underrecognition of the scope of DHTs means the true number of systems requiring assurance is likely much higher than reported in this study; therefore, the compliance gap is likely even wider.

The challenges in obtaining data for this study are, in themselves, a significant finding that will be explored further in a subsequent publication. Of the 239 organizations, 35 (14.6%) did not respond, 36 (15.1%) could only provide partial data, and 25 (10.5%) could not respond—either claiming they did not hold the information or claiming section 12 cost and time exemptions under the act due to information not being held in an “easily accessible format” or there being “no central register for clinical systems.” In these organizations, the scale of unassessed risk appears not to be quantified, potentially not recorded on risk registers, or actively managed. These responses may indicate a culture of organizational indifference to digital clinical safety.

Despite these widespread issues, it is crucial to note that full compliance is achievable. In total, 13 (6.4%) of the 204 organizations in our sample reported full compliance for all their deployed DHTs, with the highest number of DHTs in a fully compliant trust being 50. This demonstrates that adherence to the standards is possible for organizations managing a considerable number of systems, which applies to more than half (n=111, 54.4%) of the organizations in our sample.

### Implications for the Digital Clinical Safety Standards Review

NHS England is currently reviewing both DCB0129 and DCB0160 standards [38], citing the acceleration of digital transformation and the emergence of new technologies such as AI. The introduction of complex technologies, including generative AI with its known safety and data risks, into an environment where current compliance is already below 18% is a recipe for potential disaster.

New standards, while welcome, will have minimal impact without robust mechanisms for monitoring and enforcement. This study highlights critical failures that the revised standards must seek to address as follows:

- The current self-regulatory model is clearly failing. The new standards must establish external accountability for compliance.
- A central, preferably public database for all DCB0129 CSCRs is essential. For DCB0160, mechanisms are needed to enable trusts to share assessments, learn from each other’s experiences with the same DHTs, and rapidly notify other CSOs of local incidents to prevent harm from scaling.
- The updated standards must include provisions to support NHS trusts in meeting compliance, such as training more CSOs, advocating for dedicated CSO roles and time, and providing platforms for collaborative work on system reviews.
- The current standards lack a definition or audit mechanism for the *quality* of safety cases, and the CSO qualification can be achieved with minimal instruction. Future standards must address the quality assurance of safety documentation and CSO competency.

### Limitations

This study, while the first of its kind to provide a snapshot of DHT compliance in the NHS in England, has several limitations. The data were self-reported via FoI requests, and, as evidenced by the variability of responses, organizations may not have accurately reported system numbers due to differing interpretations or incomplete records. We also lacked data on the complexity and scale of individual DHTs reported, so we could not estimate the scale of clinical risk posed by their lack of assurance.

This study did not assess the quality of the safety cases that were reported as present; we acknowledge that the presence of either safety standard is not a reliable indicator of high-quality risk management practices. Examples exist where safety standards have seemingly been met but did not achieve their intended purpose—for instance, where they consisted of minimally adapted templates (“boiler plate” approaches), safety cases that were not updated following system changes, or complete safety cases adopted from other organizations and not tailored to the local context.

The quality of the safety documentation is likely driven primarily by the quality of the CSO undertaking it. In our experience, effective CSOs all have 3 shared qualities: a good understanding of the technology, sufficient contextual clinical experience through which they model how technology interacts with clinical and nonclinical pathways, and confidence. The latter is particularly important as CSOs are often required to challenge senior decision makers during sensitive periods, such as development sprints or procurement processes, where the CSO must, as data protection officers do, maintain their independence and prevent being unduly influenced, ensuring they act in the interest of the patients they are ultimately trying to protect.

We know from our practice that this is more difficult when CSOs are inexperienced, both in clinical safety and clinical experience, and it is further confounded by the minimal instruction and training (typically 9-12 hours) that CSOs receive before being qualified, much of it being self-led through online modules.

Improved training and assessment, a tiered approach to eligibility to undertake clinical safety assessments based on product complexity and CSO experience, and professionalization of the role would go a long way to bringing the recognition this critical role needs to bring it on par with other safety-critical roles, such as that of the data protection officer.

Despite these limitations, we do assert that the absence of safety standards is an indicator of poor practice.

Finally, our scope did not extend to primary care or social care settings where these standards also apply.

### Conclusions

The vision of an NHS transformed by digital technology hinges on the foundational principle of patient safety. Our findings indicate that the interpretation and application of digital clinical safety standards are alarmingly inconsistent across England. More than 10,000 DHTs are operating without documented safety assurance, a significant compliance debt representing an unquantifiable risk of harm to both patients and organizations. To truly harness the benefits of digital health, a concerted and systematic shift toward robust governance, accountability, and a culture that prioritizes digital clinical safety is urgently required as more complex technologies become further embedded in NHS care.

## Appendix

Multimedia Appendix 1 Freedom of information request.

Multimedia Appendix 2 The list of National Health Service organizations.

## Abbreviations

AI: artificial intelligence
CSCR: clinical safety case report
CSO: clinical safety officer
DCB: Data Coordination Board
DHT: digital health technology
FoI: freedom of information
ICB: integrated care board
NHS: National Health Service
STROBE: Strengthening the Reporting of Observational Studies in Epidemiology

## Data Availability

The dataset generated or analyzed during this study are not publicly available due to the reputationally sensitive nature of the findings for the organizations involved, individual results from each should be available and published on the freedom of information publication scheme or disclosure logs of each organization, respectively; this is normally available and accessible through their individual websites.
