# Supplementary material for: Digital Health Technology Compliance With Clinical Safety Standards In the National Health Service in England: National Cross-Sectional Study
Source: J Med Internet Res. 2025 Oct 31;27:e80076. doi: 10.2196/80076 (PMC12619009; doi:10.2196/80076)
Supplement: Multimedia Appendix 1 [file jmir_v27i1e80076_app1.doc]

# Appendix A

Subject: FOI Request – Clinical Safety of Health IT Systems

Dear FOI Team at <organisation>,

I am writing to request information under the Freedom of Information Act 2000 regarding the use of Health IT Systems in your organisation.

For the purposes of this FOI, Health IT Systems are defined as “[a] Product used to provide electronic information for health or social care purposes. The product may be hardware, software or a combination.”

Please note that this definition encompasses any hardware or software product used within the organisation. It does not have to be used for a clinical purpose (i.e., it may be clerical) and applies to both non-medical and medical devices (the latter as defined by UKMDR2002).

This definition is taken from the following sources:

- [DCB0129: Clinical Risk Management: its Application in the Manufacture of Health IT Systems - NHS England Digital](https://digital.nhs.uk/data-and-information/information-standards/information-standards-and-data-collections-including-extractions/publications-and-notifications/standards-and-collections/dcb0129-clinical-risk-management-its-application-in-the-manufacture-of-health-it-systems)
- [DCB0160: Clinical Risk Management: its Application in the Deployment and Use of Health IT Systems - NHS England Digital](https://digital.nhs.uk/data-and-information/information-standards/information-standards-and-data-collections-including-extractions/publications-and-notifications/standards-and-collections/dcb0160-clinical-risk-management-its-application-in-the-deployment-and-use-of-health-it-systems)

**Requested Information:**

1. The **total number of Health IT systems** currently in use by the organisation.
2. The number of Health IT systems currently in use by the organisation that have **BOTH DCB0129 (from the manufacturer) AND DCB0160 (from the deploying organisation) Clinical Safety Case Reports**.
3. The number of Health IT systems currently in use by the organisation that **have a valid DCB0129 Clinical Safety Case Report from the manufacturer ONLY** (i.e. have a DCB0129 report but NOT a DCB0160 report).
4. The number of Health IT systems currently in use by the organisation that **have a valid DCB0160 Clinical Safety Case Report from the deploying organisation ONLY** (i.e. have a DCB0160 report but NOT a DCB0129 report from the manufacturer).
5. The **total Whole Time Equivalent (WTE) of Clinical Safety Officers** who are currently actively working on DCB0129/DCB0160 implementation within the organisation.

**Preferred Response Format**

We would appreciate it if you could respond with numbers against each of the requested items above, or provide the information formatted in a table for clarity.

**Timeframe**

The information requested relates to the current situation within the organisation as of the date you receive or action this FOI request.

**Response Deadline**

Please provide this information within 20 working days, as required by the Freedom of Information Act 2000.

If you require any clarification or have any questions about this request, please feel free to contact me using the details below.

Yours faithfully,
