# Supplementary material for: Digital Health Technology Compliance With Clinical Safety Standards In the National Health Service in England: National Cross-Sectional Study
Source: J Med Internet Res. 2025 Oct 31;27:e80076. doi: 10.2196/80076 (PMC12619009; doi:10.2196/80076)
Supplement: Multimedia Appendix 2 [file jmir_v27i1e80076_app2.doc]

# Appendix B

| **1** | Airedale & Bradford NHS Foundation Trust |
| --- | --- |
| **2** | Alder Hey Children's NHS Foundation Trust |
| **3** | Ashford and St Peter's Hospitals NHS Foundation Trust |
| **4** | Avon and Wiltshire Mental Health Partnership NHS Trust |
| **5** | Barking, Havering and Redbridge University Hospitals NHS Trust |
| **6** | North London NHS Foundation Trust |
| **7** | Barnsley Hospital NHS Foundation Trust |
| **8** | Barts Health NHS Trust |
| **9** | Bedfordshire Hospitals NHS Foundation Trust |
| **10** | Berkshire Healthcare NHS Foundation Trust |
| **11** | Birmingham and Solihull Mental Health NHS Foundation Trust |
| **12** | Birmingham Community Healthcare NHS Foundation Trust |
| **13** | Birmingham Women's and Children's NHS Foundation Trust |
| **14** | Black Country Healthcare NHS Foundation Trust |
| **15** | Blackpool Teaching Hospitals NHS Foundation Trust |
| **16** | Bolton NHS Foundation Trust |
| **17** | Bradford District Care NHS Foundation Trust |
| **18** | Bradford Teaching Hospitals NHS Foundation Trust |
| **19** | Bridgewater Community Healthcare NHS Foundation Trust |
| **20** | Buckinghamshire Healthcare NHS Trust |
| **21** | Calderdale and Huddersfield NHS Foundation Trust |
| **22** | Cambridge University Hospitals NHS Foundation Trust |
| **23** | Cambridgeshire and Peterborough NHS Foundation Trust |
| **24** | Cambridgeshire Community Services NHS Trust |
| **25** | Central and North West London NHS Foundation Trust |
| **26** | Central London Community Healthcare NHS Trust |
| **27** | Chelsea and Westminster Hospital NHS Foundation Trust |
| **28** | Cheshire and Wirral Partnership NHS Foundation Trust |
| **29** | Chesterfield Royal Hospital NHS Foundation Trust |
| **30** | Cornwall Partnership NHS Foundation Trust |
| **31** | County Durham and Darlington NHS Foundation Trust |
| **32** | Coventry and Warwickshire Partnership NHS Trust |
| **33** | Croydon Health Services NHS Trust |
| **34** | Cumbria, Northumberland, Tyne and Wear NHS Foundation Trust |
| **35** | Dartford and Gravesham NHS Trust |
| **36** | Derbyshire Community Health Services NHS Foundation Trust |
| **37** | Derbyshire Healthcare NHS Foundation Trust |
| **38** | Devon Partnership NHS Trust |
| **39** | Doncaster and Bassetlaw Teaching Hospitals NHS Foundation Trust |
| **40** | Dorset County Hospital NHS Foundation Trust |
| **41** | Dorset Healthcare University NHS Foundation Trust |
| **42** | The Dudley Group NHS Foundation Trust |
| **43** | East Cheshire NHS Trust |
| **44** | East and North Hertfordshire NHS Trust |
| **45** | East Kent Hospitals University NHS Foundation Trust |
| **46** | East Lancashire Hospitals NHS Trust |
| **47** | East London NHS Foundation Trust |
| **48** | East Midlands Ambulance Service NHS Trust |
| **49** | East of England Ambulance Service NHS Trust |
| **50** | East Suffolk and North Essex NHS Foundation Trust |
| **51** | East Sussex Healthcare NHS Trust |
| **52** | Epsom and St Helier University Hospitals NHS Trust |
| **53** | Essex Partnership University NHS Foundation Trust |
| **54** | Frimley Health NHS Foundation Trust |
| **55** | Gateshead Health NHS Foundation Trust |
| **56** | George Eliot Hospital NHS Trust |
| **57** | Gloucestershire Health and Care NHS Foundation Trust |
| **58** | Gloucestershire Hospitals NHS Foundation Trust |
| **59** | Great Ormond Street Hospital for Children NHS Foundation Trust |
| **60** | Great Western Hospitals NHS Foundation Trust |
| **61** | Greater Manchester Mental Health NHS Foundation Trust |
| **62** | Guy's and St Thomas’s NHS Foundation Trust |
| **63** | Hampshire Hospitals NHS Foundation Trust |
| **64** | Harrogate and District NHS Foundation Trust |
| **65** | Hertfordshire Community NHS Trust |
| **66** | Hertfordshire Partnership University NHS Foundation Trust |
| **67** | Homerton University Hospital NHS Foundation Trust |
| **68** | Hounslow and Richmond Community Healthcare NHS Trust |
| **69** | Hull University Teaching Hospitals NHS Trust |
| **70** | Humber Teaching NHS Foundation Trust |
| **71** | Imperial College Healthcare NHS Trust |
| **72** | Isle of Wight NHS Trust |
| **73** | James Paget University Hospitals NHS Foundation Trust |
| **74** | Kent and Medway NHS and Social Care Partnership Trust |
| **75** | Kent Community Health NHS Foundation Trust |
| **76** | Kettering General Hospital NHS Foundation Trust |
| **77** | King's College Hospital NHS Foundation Trust |
| **78** | Kingston and Richmond NHS Foundation Trust |
| **79** | Lancashire and South Cumbria NHS Foundation Trust |
| **80** | Lancashire Teaching Hospitals NHS Foundation Trust |
| **81** | Leeds and York Partnership NHS Foundation Trust |
| **82** | Leeds Community Healthcare NHS Trust |
| **83** | Leeds Teaching Hospitals NHS Trust |
| **84** | Leicestershire Partnership NHS Trust |
| **85** | Lincolnshire Community Health Services NHS Trust |
| **86** | Lincolnshire Partnership NHS Foundation Trust |
| **87** | Liverpool Heart and Chest Hospital NHS Foundation Trust |
| **88** | Liverpool Women's NHS Foundation Trust |
| **89** | London Ambulance Service NHS Trust |
| **90** | London North West University Healthcare NHS Trust |
| **91** | Maidstone and Tunbridge Wells NHS Trust |
| **92** | Manchester University NHS Foundation Trust |
| **93** | Medway NHS Foundation Trust |
| **94** | Mersey Care NHS Foundation Trust |
| **95** | Mid and South Essex NHS Foundation Trust |
| **96** | Mid Cheshire Hospitals NHS Foundation Trust |
| **97** | Mid Yorkshire Hospitals NHS Trust |
| **98** | Milton Keynes University Hospital NHS Foundation Trust |
| **99** | Moorfields Eye Hospital NHS Foundation Trust |
| **100** | Norfolk and Norwich University Hospitals NHS Foundation Trust |
| **101** | Norfolk and Suffolk NHS Foundation Trust |
| **102** | North Bristol NHS Trust |
| **103** | North Cumbria Integrated Care NHS Foundation Trust |
| **104** | North East Ambulance Service NHS Foundation Trust |
| **105** | North East London NHS Foundation Trust |
| **106** | North Staffordshire Combined Healthcare NHS Trust |
| **107** | North Tees and Hartlepool NHS Foundation Trust |
| **108** | North West Ambulance Service NHS Trust |
| **109** | North West Anglia NHS Foundation Trust |
| **110** | Northampton General Hospital NHS Trust |
| **111** | Northamptonshire Healthcare NHS Foundation Trust |
| **112** | Northern Care Alliance NHS Foundation Trust |
| **113** | Northern Lincolnshire and Goole NHS Foundation Trust |
| **114** | Northumbria Healthcare NHS Foundation Trust |
| **115** | Nottingham University Hospitals NHS Trust |
| **116** | Nottinghamshire Healthcare NHS Foundation Trust |
| **117** | Oxford Health NHS Foundation Trust |
| **118** | Oxford University Hospitals NHS Foundation Trust |
| **119** | Oxleas NHS Foundation Trust |
| **120** | Pennine Care NHS Foundation Trust |
| **121** | Portsmouth Hospitals University NHS Trust |
| **122** | Queen Victoria Hospital NHS Foundation Trust |
| **123** | Royal Berkshire NHS Foundation Trust |
| **124** | Royal Cornwall Hospitals NHS Trust |
| **125** | Royal Devon University Healthcare NHS Foundation Trust |
| **126** | Royal Free London NHS Foundation Trust |
| **127** | University Hospitals of Liverpool Group |
| **128** | Royal Papworth Hospital NHS Foundation Trust |
| **129** | Royal Surrey NHS Foundation Trust |
| **130** | Royal United Hospitals Bath NHS Foundation Trust |
| **131** | Royal Wolverhampton NHS Trust |
| **132** | Salisbury NHS Foundation Trust |
| **133** | Sheffield Children's NHS Foundation Trust |
| **134** | Sheffield Health and Social Care NHS Foundation Trust |
| **135** | Sheffield Teaching Hospitals NHS Foundation Trust |
| **136** | Sherwood Forest Hospitals NHS Foundation Trust |
| **137** | Shropshire Community Health NHS Trust |
| **138** | Hampshire and Isle of Wight Healthcare NHS Foundation Trust |
| **139** | Somerset NHS Foundation Trust |
| **140** | South Central Ambulance Service NHS Foundation Trust |
| **141** | South East Coast Ambulance Service NHS Foundation Trust |
| **142** | South London and Maudsley NHS Foundation Trust |
| **143** | South Tees Hospitals NHS Foundation Trust |
| **144** | South Tyneside and Sunderland NHS Foundation Trust |
| **145** | South Warwickshire NHS Foundation Trust |
| **146** | South West London and St George's Mental Health NHS Trust |
| **147** | South Western Ambulance Service NHS Foundation Trust |
| **148** | Mersey and West Lancashire Teaching Hospitals NHS Trust |
| **149** | St George's University Hospitals NHS Foundation Trust |
| **150** | Stockport NHS Foundation Trust |
| **151** | Surrey and Borders Partnership NHS Foundation Trust |
| **152** | Surrey and Sussex Healthcare NHS Trust |
| **153** | Sussex Community NHS Foundation Trust |
| **154** | Sussex Partnership NHS Foundation Trust |
| **155** | Tameside and Glossop Integrated Care NHS Foundation Trust |
| **156** | Tavistock and Portman NHS Foundation Trust |
| **157** | The Christie NHS Foundation Trust |
| **158** | The Clatterbridge Cancer Centre NHS Foundation Trust |
| **159** | The Hillingdon Hospitals NHS Foundation Trust |
| **160** | The Newcastle upon Tyne Hospitals NHS Foundation Trust |
| **161** | The Princess Alexandra Hospital NHS Trust |
| **162** | The Queen Elizabeth Hospital King's Lynn NHS Foundation Trust |
| **163** | The Robert Jones and Agnes Hunt Orthopaedic Hospital NHS Foundation Trust |
| **164** | The Rotherham NHS Foundation Trust |
| **165** | The Royal Marsden NHS Foundation Trust |
| **166** | The Royal Orthopaedic Hospital NHS Foundation Trust |
| **167** | The Walton Centre NHS Foundation Trust |
| **168** | Torbay and South Devon NHS Foundation Trust |
| **169** | United Lincolnshire Hospitals NHS Trust |
| **170** | University College London Hospitals NHS Foundation Trust |
| **171** | University Hospital Southampton NHS Foundation Trust |
| **172** | University Hospitals Birmingham NHS Foundation Trust |
| **173** | University Hospitals Bristol and Weston NHS Foundation Trust |
| **174** | University Hospitals Coventry and Warwickshire NHS Trust |
| **175** | University Hospitals Dorset NHS Foundation Trust |
| **176** | University Hospitals of Derby and Burton NHS Foundation Trust |
| **177** | University Hospitals of Leicester NHS Trust |
| **178** | University Hospitals of Morecambe Bay NHS Foundation Trust |
| **179** | University Hospitals of North Midlands NHS Trust |
| **180** | University Hospitals Plymouth NHS Trust |
| **181** | University Hospitals Sussex NHS Foundation Trust |
| **182** | Walsall Healthcare NHS Trust |
| **183** | Warrington and Halton Teaching Hospitals NHS Foundation Trust |
| **184** | West Hertfordshire Teaching Hospitals NHS Trust |
| **185** | West London NHS Trust |
| **186** | West Midlands Ambulance Service University NHS Foundation Trust |
| **187** | West Suffolk NHS Foundation Trust |
| **188** | Wirral Community Health and Care NHS Foundation Trust |
| **189** | Wirral University Teaching Hospital NHS Foundation Trust |
| **190** | Worcestershire Acute Hospitals NHS Trust |
| **191** | Herefordshire and Worcestershire Health and Care NHS Trust |
| **192** | Wrightington, Wigan and Leigh Teaching Hospitals NHS Foundation Trust |
| **193** | Wye Valley NHS Trust |
| **194** | York and Scarborough Teaching Hospitals NHS Foundation Trust |
| **195** | Yorkshire Ambulance Service NHS Trust |
| **196** | Countess of Chester Hospitals NHS Foundation Trust |
| **197** | Tees, Esk and Wear Valley NHS FT |
| **198** | Bath and North East Somerset, Swindon and Wiltshire ICB |
| **199** | Bedfordshire, Luton and Milton Keynes ICB |
| **200** | Birmingham and Solihull ICB |
| **201** | Black Country ICB |
| **202** | Bristol, North Somerset and South Gloucestershire ICB |
| **203** | Buckinghamshire, Oxfordshire and Berkshire West ICB |
| **204** | Cambridgeshire and Peterborough ICB |
| **205** | Cheshire and Merseyside ICB |
| **206** | Cornwall and the Isles of Scilly ICB |
| **207** | Coventry and Warwickshire ICB |
| **208** | Derby and Derbyshire ICB |
| **209** | Devon ICB |
| **210** | Dorset ICB |
| **211** | Frimley ICB |
| **212** | Gloucestershire ICB |
| **213** | Greater Manchester ICP |
| **214** | Hampshire and Isle of Wight ICB |
| **215** | Herefordshire and Worcestershire ICB |
| **216** | Hertfordshire and West Essex ICB |
| **217** | Humber and North Yorkshire ICB |
| **218** | Kent and Medway ICB |
| **219** | Lancashire and South Cumbria ICB |
| **220** | Leicester, Leicestershire and Rutland ICB |
| **221** | Lincolnshire ICB |
| **222** | Mid and South Essex ICB |
| **223** | Norfolk and Waveney ICB |
| **224** | North Central London ICB |
| **225** | North East and North Cumbria ICB |
| **226** | North East London ICB |
| **227** | North West London ICB |
| **228** | Northamptonshire ICB |
| **229** | Nottingham and Nottinghamshire ICB |
| **230** | Shropshire, Telford and Wrekin ICB |
| **231** | Somerset ICB |
| **232** | South East London ICB |
| **233** | South West London ICB |
| **234** | South Yorkshire ICB |
| **235** | Staffordshire and Stoke-on-Trent ICB |
| **236** | Suffolk and North East Essex ICB |
| **237** | Surrey Heartlands |
| **238** | Sussex ICB |
| **239** | West Yorkshire ICB |
